# Vaccination against the HDL receptor of *S. japonicum* inhibits egg embryonation and prevents fatal hepatic complication in rabbit model

**DOI:** 10.1371/journal.pntd.0011749

**Published:** 2023-11-29

**Authors:** Jifeng Zhu, Lina Zhang, Zechao Xue, Zilüe Li, Chun Wang, Fanyan Chen, Yalin Li, Yang Dai, Yonghua Zhou, Sha Zhou, Xiaojun Chen, Kuniko Okumura-Noji, Rui Lu, Shinji Yokoyama, Chuan Su

**Affiliations:** 1 National Vaccine Innovation Platform, Nanjing Medical University, Nanjing, Jiangsu, China; 2 Jiangsu Key Laboratory of Pathogen Biology, Nanjing Medical University, Nanjing, Jiangsu, China; 3 Department of Pathogen Biology and Immunology, School of Basic Medical Sciences, Nanjing Medical University, Nanjing, Jiangsu, China; 4 Department of Blood Transfusion, Henan Provincial People’s Hospital, Zhengzhou, Henan, China; 5 Department of Blood Transfusion of Central China Fuwai Hospital, Central China Fuwai Hospital of Zhengzhou University, Zhengzhou, Henan, China; 6 Jiangsu Institute of Parasitic Diseases, Wuxi, Jiangsu, China; 7 Food and Nutritional Sciences, Chubu University, Kasugai, Japan; Instituto de Salud Carlos III, SPAIN

## Abstract

**Background:**

Schistosomiasis is one of the most important neglected tropical infectious diseases to overcome and the primary cause of its pathogenesis is ectopic maturation of the parasite eggs. Uptake of cholesteryl ester from the host high-density lipoprotein (HDL) is a key in this process in *Schistosoma japonicum* and CD36-related protein (CD36RP) has been identified as the receptor for this reaction. Antibody against the extracellular domain of CD36RP (Ex160) efficiently blocked the HDL cholesteryl ester uptake and the egg embryonation *in vitro*. However, whether Ex160 immunization could efficiently raise proper antibody responses to sufficiently block HDL cholesteryl ester uptake and the egg embryonation to protect host *in vivo* is very interesting but unknown.

**Methodology/Principal findings:**

In this study, rabbits were immunized with the recombinant Ex160 peptide (rEx160) to evaluate its anti-pathogenic vaccine potential. Immunization with rEx160 induced consistent anti-Ex160 IgG antibody and significant reduction in development of the liver granulomatosis lesions associated with suppressed intrahepatic maturation of the schistosome eggs. The immunization with rEx160 rescued reduction of serum HDL by the infection without changing its size distribution, being consistent with interference of the HDL lipid uptake by the parasites or their eggs by antibody against Ex160 in *in vitro* culture.

**Conclusions/Significance:**

The results demonstrated that vaccination strategy against nutritional supply pathway of the parasite is effective for reducing its pathogenesis.

## Author summary

Schistosomiasis remains a major neglected tropical disease affecting over 200 million people worldwide. Human and animals infected with *Schistosoma japonicum* (*S*. *japonicum*) or *Schistosoma mansoni* develop a severe liver pathology which arises when schistosome eggs lodged in the liver. The secretions from the embolized mature eggs elicits the host’s granulomatous cell-mediated immune response, which progresses to irreversible fibrosis and lethal liver pathogenesis. The schistosomes obtain nutrients from the host blood by using nonspecific and specific transport via their body surface and alimentary tracts, and CD36-related protein (CD36RP) was identified in both adult worms and eggs of *S*. *japonicum*, which mediates the uptake of cholesteryl ester from the host plasma high-density lipoproteins and facilitates the maturation of the eggs. In this study, we found that immunization with extracellular domain of CD36RP (Ex160) suppressed the egg maturation of schistosomes and reduced liver injury in *S*. *japonicum* infected rabbits. Ex160 may serve as a candidate for the antigen for vaccination against schistosomal infection.

## Introduction

Schistosomiasis remains one of the most prevalent, insidious, and serious tropical parasitic diseases in 78 countries, with an estimated 251 million people requiring treatment in 2021, mainly by three species of schistosomes: *Schistosoma mansoni* (*S*. *mansoni*) in Africa and South America, *Schistosoma haematobium* in Africa and the Middle East and *Schistosoma japonicum* (*S*. *japonicum*) in Asia such as China, Indonesia, the Philippines, Indochina peninsula, and previously in Japan as well [1]. The parasites reside in the visceral blood vessels and lay fertilized eggs excreted for their normal life cycle. However, some of them remain plugged in the host body and result in ectopic maturation for embryonation, which become a major factor of the pathogenesis for serious chronic inflammation [2–4]. *S*. *mansoni* and *S*. *japonicum* eggs primarily cause liver and intestinal damage, particularly liver damage. Intrahepatic deposition of the eggs causes a continuous antigenic stimulation, subsequent liver fibrosis, portal hypertension, hemorrhage and eventually costs life [3,4].

Studies have proven that secretions from the embolized eggs during ectopic maturation/embryonation in the tissues initiate strong inflammatory granulomatous response [2,5,6] and drive the serious and sometimes lethal liver pathogenesis [3,4]. The eggs of *S*. *japonicum* are laid with ovum inside into the host circulation and develop embryonation to miracidia in approximately the next ten days [7,8]. This process requires acquisition of nutrients such as lipids, amino acids, glucose and else from the host blood plasma [8].

Cholesteryl ester (CE) in HDL of the host blood plasma seems one of the essential elements of immature eggs of *S*. *japonicum* to grow and develop to mature miracidia [9–11]. The uptake of CE from human HDL is mediated by the parasite HDL receptor, CD36RP. The extracellular domain peptide of CD36RP, Ex160, is located at positions 797–1280 of the CD36RP nucleotide sequence and encodes a fragment of 160 amino acids from G249 to P408. Ex160 binds to the host HDL and facilitates the transport of CE from HDL to the parasite [9,12,13]. In *in vitro* culture, the antibody against Ex160 blocks the binding of CD36RP to human HDL and significantly suppressed the active CE uptake, which leads to inhibit the egg embryonation to miracidium [12]. Lack of cholesteryl ester transfer protein (CETP) causes accumulation of CE in HDL to make it extremely large, and this HDL seems a poor supplier of CE to the schistosome eggs for their maturation [9–11]. It is therefore hypothesized that inhibition of this HDL receptor-mediated CE uptake pathway to prevent maturation of the eggs *in vivo* would reduce the fatal hepatic complication in *S*. *japonicum* infection.

The present study showed that the pre-immunization of the rabbits with recombinant *S*. *japonicum* Ex160 peptide efficiently induced anti-Ex160 antibody and to substantially reduced hepatic granulomatosis in the *S*. *japonium*-infected rabbits apparently by preventing the egg maturation. Our results indicated that recombinant *S*. *japonicum* Ex160 is a promising candidate for the antigen for vaccination against schistosomal infection.

## Materials and methods

### Ethics statement

The animal studies were carried out in strict accordance with the recommendations in the Guide for the Care and Use of Laboratory Animals of the Ministry of Science and Technology of the People’s Republic of China. The animal protocol was approved by the Committee on the Ethics of Animal Experiments of Parasitic Disease Prevention and Research Institute of Jiangsu Province (Permit Number: IACUC-JIPD-2017011).

### Production of the antigen peptide

#### Strains and plasmids

*Escherichia coli* (*E*. *coli*) DH5α was used as the host strain for recombinant plasmid preparation. *E*. *coli* BL21 was used as an expression host. The expression vector used was plasmid pQE-30, which contains the Lac promoter and allows expression of recombinant protein fused to the C-terminus of histidine (His).

#### Gene synthesis and cloning

The Ex160-cDNA was synthesized with codon optimisation (S1 Data) for procaryotic expression (GenScript Biotech Co., Ltd., Nanjing, China) and cloned into BamHI/HindIII restriction sites in the same reading frame with the His tag in the pQE-30 vector. The recombinant plasmid was transformed into *E*. *Coli* DH5α. The plasmid was extracted from the bacteria using a plasmid purification kit (Tiangen Biotech Co., Ltd., Beijing, China), and digested by BamHI/HindIII enzymes (NEB, Beverly, MA, USA) to confirm ligation of this gene into the pQE-30 plasmid. Agarose gel electrophoresis was performed to confirm the correct insertion of the target gene.

#### Expression of recombinant Ex160-His protein in *E*. *coli*

The plasmid pQE-30-Ex160 was transformed into *E*. *coli* BL21. Culture of recombinant *E*. *coli* was performed in shake flasks. Luria broth medium (LB, Oxoid Ltd., Basingstoke, UK) containing 100 μg/ml of ampicillin (Thermo Fisher Scientific, MA, USA) was inoculated with the recombinant *E*. *coli* and incubated in a shaker incubator (Zhicheng, Shanghai, China) at 37°C, 200 rpm for 14 h. Overnight cultures were diluted 100-fold and grown at 37°C until an OD 600 nm of 0.8. A final concentration of 0.5 mM Isopropyl β-D-thiogalactopyranoside (IPTG, Thermo Fisher Scientific) was added for 4-h induction of protein expression. Cultures were then centrifuged at 4°C, 9000 rpm for 30 min and the pellets were lysed using lysis buffer (50 mM Tris-HCl, pH 8.0, 300 mM NaCl). After sonication at 500 w, cell pellets were spun down at 9000 rpm, 4°C for 30 min, the supernatant was discarded. The inclusion body pellet was solubilized in denature buffer (50 mM Tris-HCl, pH 8.0, 8 M urea) by sonication. The sample was spun at 4°C, 9000 rpm for 30 min. And the supernatant was used for further purification.

#### Purification of recombinant proteins

The recombinant protein, with an affinity tag of six consecutive histidine residues, was purified by affinity chromatography with cOmplete His-Tag purification resin (Roche Diagnostics, Indianapolis, IN, USA) following the manufacturer’s instructions. Fractions of interest were pooled and dialyzed 10 times against 2 L dialysis buffer (1× PBS+0.5% Sodium Lauroyl Sarcosine) at 4°C. Intervals between changes of buffers were 2 h. Dialyzed proteins are pooled and concentrated using a centrifugal filter unit (MWCO 10KD, Millipore, Billerica, MA, USA). All prepared samples were rendered sterile by filtration (0.22 μm, Millipore).

#### SDS-PAGE analysis of recombinant protein

Samples were resolved by SDS-PAGE, (15% w/v separating gel) using a Mini-PROTEAN Tetra Electrophoresis System (Bio-Rad Laboratories, CA, USA). Protein calibration markers (Thermo Fisher Scientific) were used as size standards. Proteins were visualized by staining with Coomassie brilliant blue R250 (Sigma-Aldrich, Shanghai, China).

#### Western blotting analysis of recombinant protein

Protein fractions separated by SDS-PAGE were transferred to a polyvinylidene difluoride membrane (Whatman Inc., Florham Park, NJ, USA). The membrane was blocked with 5% skimmed milk in PBS+0.1% Tween 20 and probed with anti-Ex160 antibody prepared in our previous study [12]. The blots were visualized using the Pierce ECL Plus Western Blotting Substrate (Thermo Fisher Scientific), detected by the ChemiDoc Touch Imaging System (Bio-Rad Laboratories).

#### Immunization and infection experiments

Complete Freund’s Adjuvants (CFA) and incomplete Freund’s Adjuvants (IFA) were from Sigma-Aldrich. Adjuvant-protein formulation was prepared the day before vaccination following the manufacturer’s recommendation.

New Zealand white male rabbits of 4- to 5-month-old were employed for the immunization experiments. The animals were divided into 6 groups of at least 5 rabbits each, and one group was immunized with buffered (1× PBS + 0.5% Sodium Lauroyl Sarcosine)rEx160 combined with CFA by subcutaneous (SC) injection at multiple sites of the back (400 μg of rEx160/rabbit/injection), followed by two subsequent boosts at 2-week intervals with rEx160 combined with IFA. Other five groups, rabbits injected with PBS (2 groups), PBS + Adjuvant, PBS + Buffer, or Buffer + Adjuvant, were control groups as described in the results. All the rabbits except for those in one control group of blank injection of PBS were subsequently challenged percutaneously with 100 ± 2 *S*. *japonicum* cercariae 2 weeks after the last immunization.

### Antibody determination

An indirect-ELISA was used to detect anti-rEx160 IgG antibody. The antigen, 1 ng per well, was used to coat a 96-well flat bottom polystyrene High Binding plate (Corning, Acton, MA, USA) overnight at 4°C. The plates were blocked with 5% skimmed milk in PBS + 0.1% Tween 20. Antigen-specific IgG was detected using goat anti-rabbit IgG-HRP antibody (Thermo Fisher Scientific). After wash, the plates were developed by adding 3, 3’, 5, 5’-Tetramethylbenzidine (TMB) liquid substrate (Sigma-Aldrich). The reaction was stopped with 1 M H_2_SO_4_ and read on a micro-plate ELISA reader (BioStack Ready, BioTek Instruments, Vinooski, Vermont, USA) at 450 nm.

### Hematoxylin-eosin (H&E) and Sirius Red staining

Two samples from the left lobe and one sample from the right lobe of the liver, as well as a segment of the colon were taken from each rabbit and fixed in formalin. Then liver and colon samples were embedded in paraffin and cut into 3 μm-thick sections. Sections were then stained with H & E for granuloma area measurement or Sirius Red for determination of fibrotic deposition. To analyze and evaluate granuloma, at least 50 lesions were randomly chosen from each group at 5 × (liver sections) or 10 × (colon sections) objective lens and the granuloma area was measured using Zeiss AxioVision software (Zeiss, Oberkochen, Germany). To determine the intensity of fibrosis, the Sirius Red-positive area was measured at a final magnification of 100× using Zeiss AxioVision software (Zeiss). 10 images per animal were taken and analyzed using Image-Pro Plus system (Media Cybernetics Inc., Bethesda, Maryland, USA). The Sirius Red-positive staining area was produced and expressed as a percentage of the area of the field. A total fibrosis density score was also determined by dividing the image intensity by the image area. Intensity exclusion parameters were identical for each of the images captured.

### Worm pair count

Eight weeks after infection, rabbits were sacrificed. Adult worms were obtained from the portal vein by perfusion. Worms caught up in tissues near the position the portal vein was cut, were collected by hand and counted. The mesenteric veins were manually searched for adult worms. All adult worm pairs recovered from each rabbit were counted.

### *S*. *japonicum* egg counts

The whole liver was removed, washed in ice-cold PBS, blotted dry, and weighted. Two samples from the left lobe and one sample from the right lobe were obtained from each rabbit and weighed. The liver pieces from the same animal were pooled and digested in 20 ml of 5% KOH for 1 day at 37°C. The suspension was centrifuged at 3,000 rpm for 5 min, the pellet was washed and resuspended in 1 ml of PBS. Number (N) of eggs present in 10 μl aliquots was counted under microscopic observation. The number of eggs in the liver = N × 100/[total weight of three liver pieces] × [weight of the whole liver]; The number of eggs per worm pair in liver = [number of eggs in the liver] /[number of worm pairs].

### Measurement of serum levels of HDL-C

The serum levels of HDL cholesterol (HDL-C) were measured using a fully automatic biochemical analyzer (Hitachi 7600–20, Tokyo, Japan) with commercial assay kits (Roche Diagnostics, Penzberg, Germany). Subfractions of HDL were determined by a Liposorber HPLC system at the laboratory of Skylight Biotech Ltd (Akita, Japan). Seven subfractions were quantitatively calculated according to the model previously described [14].

### Determination of egg maturity

#### Maturity in the liver

Two samples from the left lobe and one sample from the right lobe were obtained from the liver of each rabbit and weighed. About 4 g of the fresh liver tissue was taken from one animal and cut into pieces before homogenization. Streptomyces proteinase (0.01%, Sigma-Aldrich) digestion was carried out for 1 h at 37°C, followed by collagenase (Type II, 0.05%, Thermo Fisher Scientific) digestion for another 1 h at 37°C. After washing with 1% NaCl twice, the pellets were collected for determination of maturation of *S*. *japonicum* eggs under microscope observation. [% maturation of eggs] = [Number of matured eggs to miracidium]/[total number of eggs examined] × 100. **Maturity in the intestine.** Colon sections stained with H & E were evaluated for the maturity of eggs in the intestine [15]. At least 40 eggs of each rabbit were observed under the microscope. [% maturation of eggs] = [Number of matured eggs to miracidium)/[total number of eggs examined] × 100.

### Statistical analysis

GraphPad prism software 5.0 (GraphPad Prism Inc., San Diego, CA, USA) was used for all statistical analysis using one-way ANOVA. p ≤ 0.05 was considered statistically significant. P values are as indicated by asterisks: *P ≤ 0.05, **P ≤ 0.01, ***P ≤ 0.001.

## Results

### Expression of recombinant Ex160-His protein

**Fig 1:**
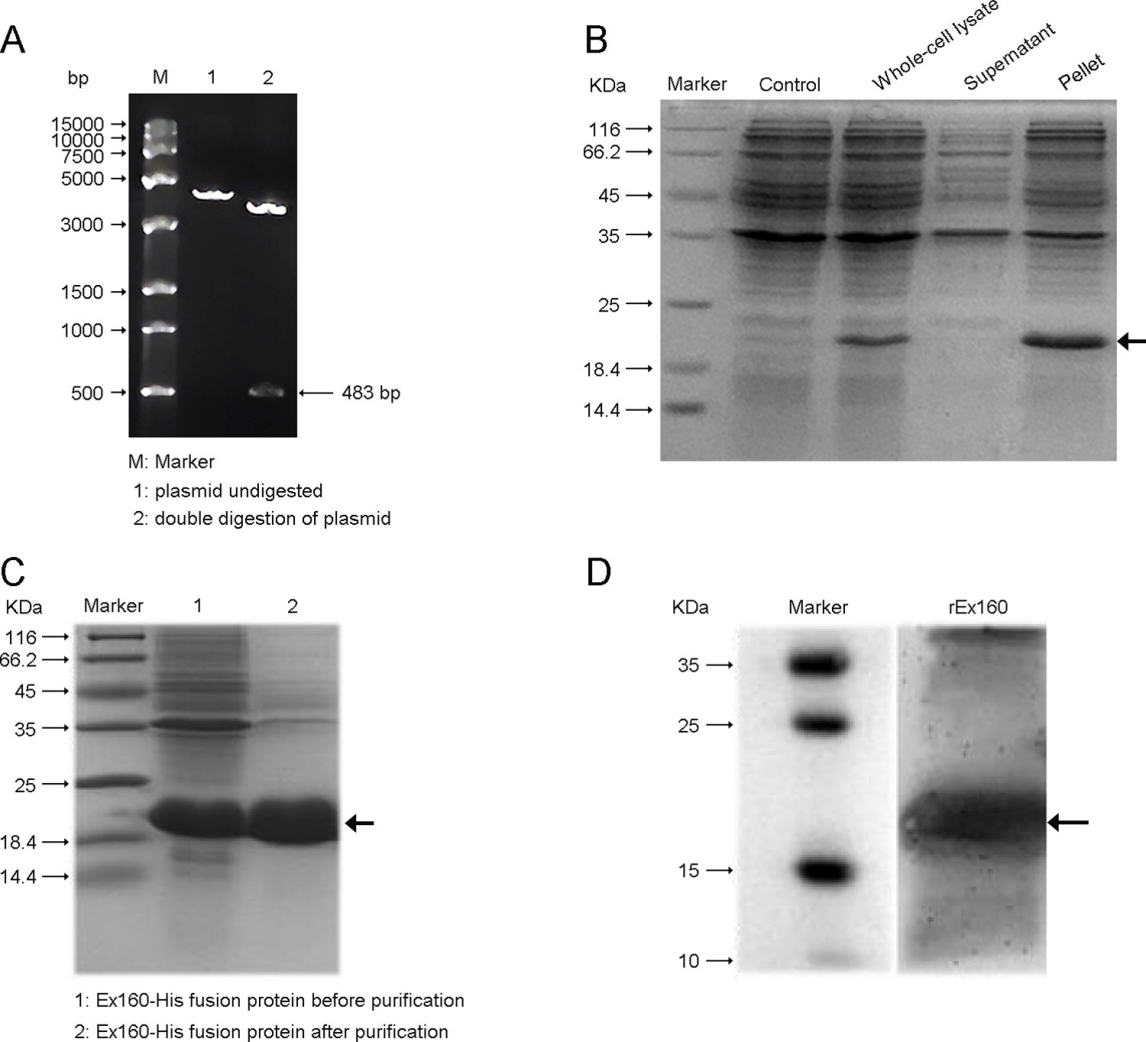
Expression of recombinant Ex160-His protein. (A) Agarose gel electrophoresis of recombinant plasmid pQE-30-Ex160 double digested with BamHI and HindIII. (B) Ex160-His expression in whole-cell lysates and subcellular fractionation of *E*. *coli*. Supernatant and pellet fractions of sonicated *E*. *coli* as well as whole-cell lysates were separated by 15% SDS-PAGE. Whole-cell lysate of competent cell was used as a control. (C) SDS-PAGE of the Ex160-His fusion protein before and after purification. (D) Western blotting of the purified Ex160-His fusion protein against anti-Ex160 antibody.

The Ex160-cDNA was synthesized and cloned into the pQE-30 vector. Digested products controlled with agarose gel electrophoresis showed a DNA band at about 483 bp (Fig 1A) indicating the insertion of the target gene was successful. Analysis of *E*. *coli* expressing Ex160-His in SDS-PAGE showed that target protein was insoluble as aggregates in inclusion bodies (Fig 1B). Purified the insoluble fractions of Ex160-His was assessed in SDS-PAGE to show the purified protein had a molecular weight of approximately 20 kDa shown in Fig 1C. Identity of the recovered Ex160-His was assured by western blotting using anti-Ex160 specific antibody [12]. It confirmed that the recombinant protein recovered from the inclusion bodies is Ex160 (rEx160) (Fig 1D). rEx160 thus purified was used for further experiments.

### Immunization and infection of rabbits

**Fig 2:**
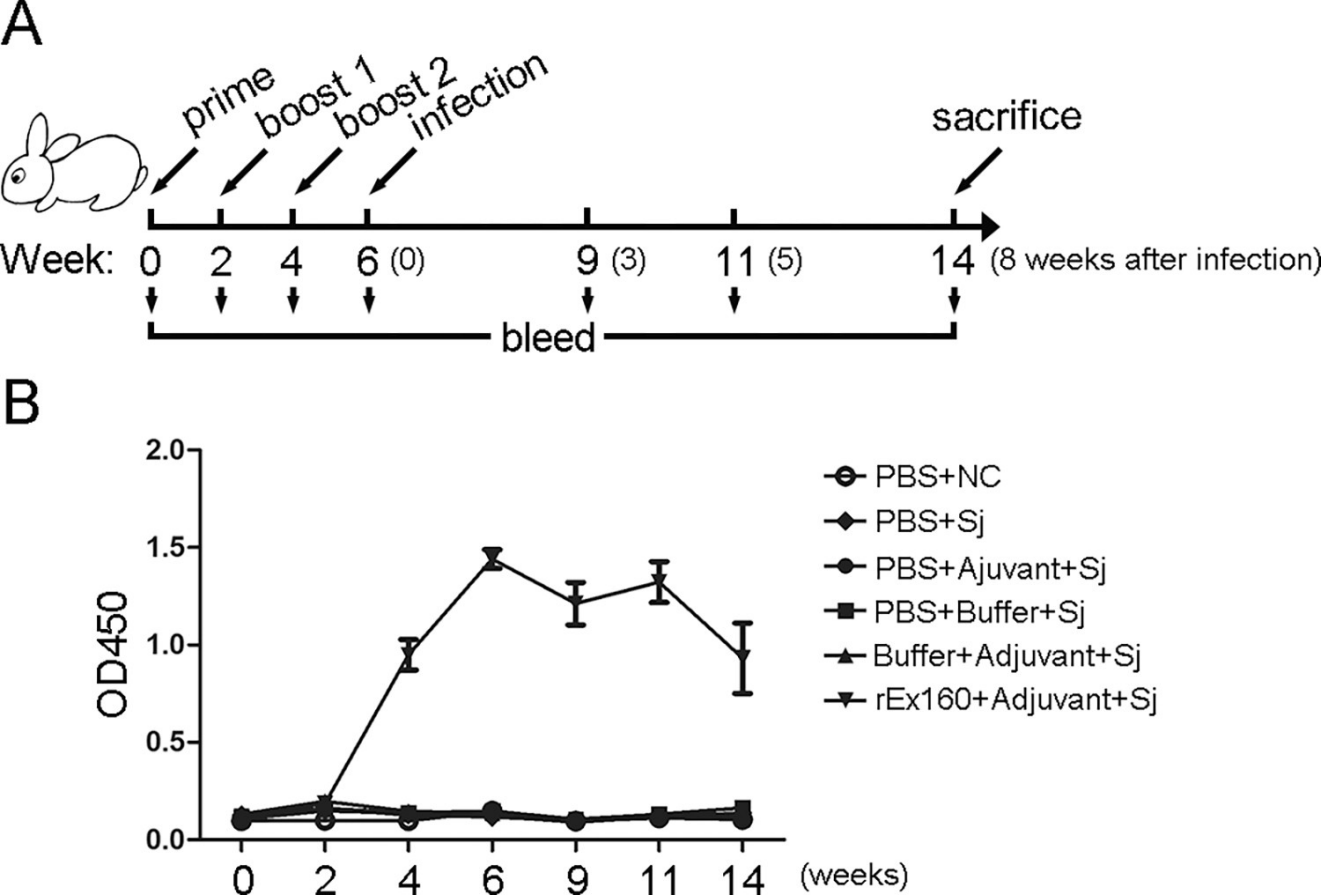
Immunization and infection of rabbits. (A) Time-schedule of animal immunization, bleeding, infection, and sacrifice. The rabbits were boosted in weeks 2 and 4. Two weeks after immunization, the rabbits were challenged with 100±2 cercariae of *S*. *japonicum*. Rabbits were sacrificed 8 weeks after schistosome infection. The bleeding strategy was as shown. (B) Rabbits were immunized with PBS, PBS+Adjuvant, PBS+Buffer, Buffer+Adjuvant or rEx160+Adjuvant and subsequently infected with *S*. *japonicum*. Rabbits injected with only PBS but without infection served as normal control (NC). Serum samples were collected at indicated time points and the titers of anti-Ex160 IgG antibody were measured by indirect-ELISA. Data were means ± SEM of samples from two independent experiments. (NC: normal control; Sj: *S*. *japonicum*).

It was determined whether immunization with rEx160 elicited specific antibodies. Sera collected from the immunized animals were tested by ELISA. The animals immunized with rEx160 showed high levels of IgG antibody against rEx160 appearing at 2 weeks after the second immunization (on week 4) while the antibody levels in the other five groups did not show significant changes. This level of antibodies was maintained until the end of the experiment (Fig 2B).

### rEx160 immunization alleviates liver pathology in the *S*. *japonicum* infected rabbits

**Fig 3:**
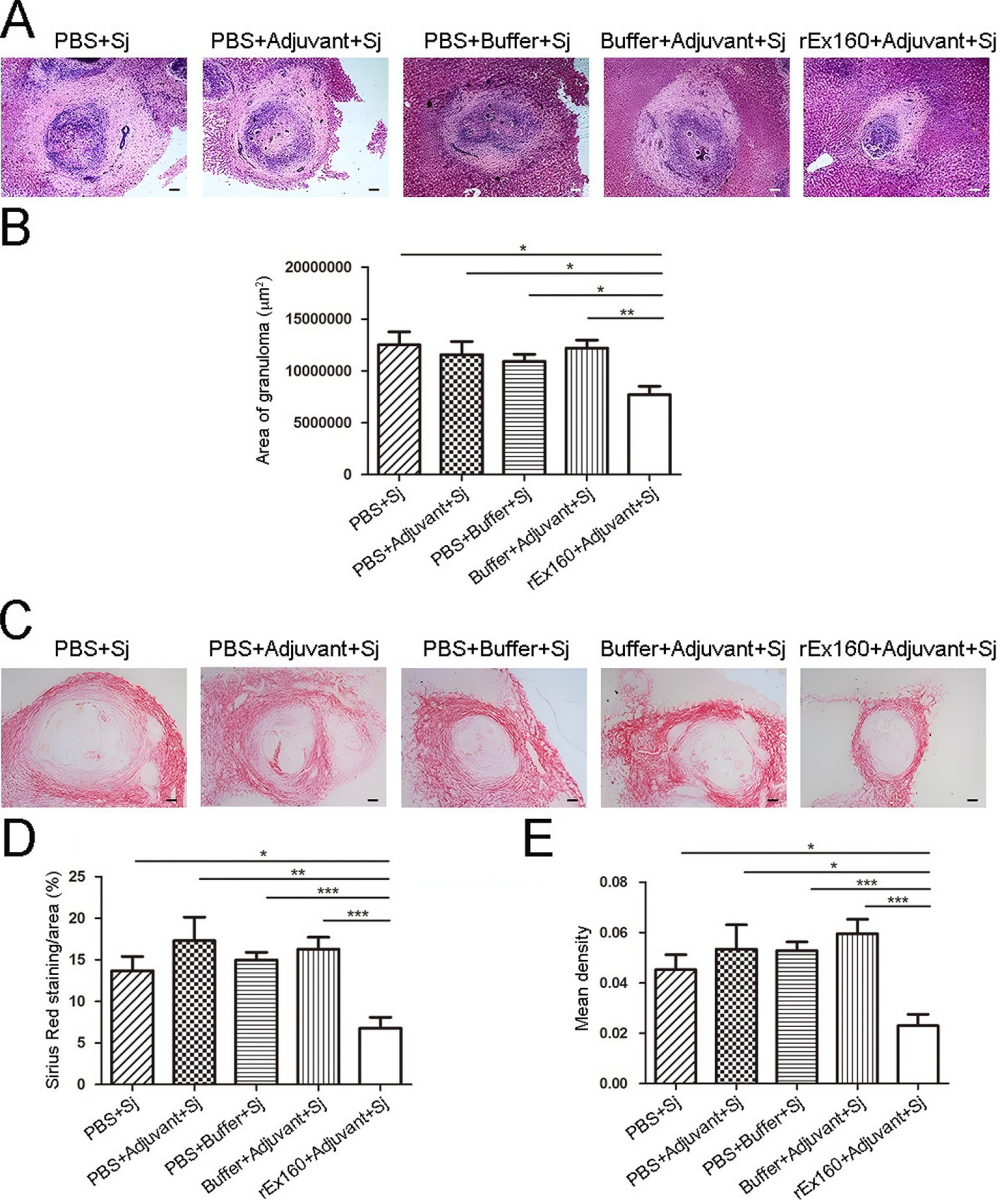
rEx160 immunization alleviates liver pathology in the *S*. *japonicum* infected rabbits. Rabbits were immunized with PBS, PBS+Adjuvant, PBS+Buffer, Buffer+Adjuvant, or rEx160+Adjuvant and subsequently infected with *S*. *japonicum*. The livers were harvested at 8 weeks post-infection and H & E or Sirius Red staining was performed in liver sections. Measurement of granuloma area was carried out. (A) Representative photos of granulomas with the original magnification of 50× (scale bar = 100 μm). (B) Quantification of granuloma area. Deposition of collagen was quantified. (C) Representative photos of Sirius Red-positive staining with the original magnification of 100× (scale bar = 50 μm). (D) Area and (E) integrated mean density of the Sirius Red-positive staining of random microscopic fields. Photos are representative of two experiments with similar results. Data were means ± SEM of samples from two independent experiments. (Sj: *S*. *japonicum*; *P≤0.05, **P≤0.01, ***P≤0.001).

The main pathologic event in schistosomiasis japonica takes place as a result of eggs deposited in the liver [2,4]. The effects of rEx160 immunization on liver pathology of the infected rabbits were therefore examined. Histological examination of the liver specimens from the rEx160 immunized rabbits collected 8 weeks after the infection (on week 14) exhibited significant reduction of the level of granulomatous response and fibrosis compared to the control groups (Fig 3). The results indicated that rEx160 immunization effectively reduced the inflammatory response in the liver induced by *S*. *japonicum* infection.

### Egg counts is not associated with pathological change in the liver

**Fig 4:**
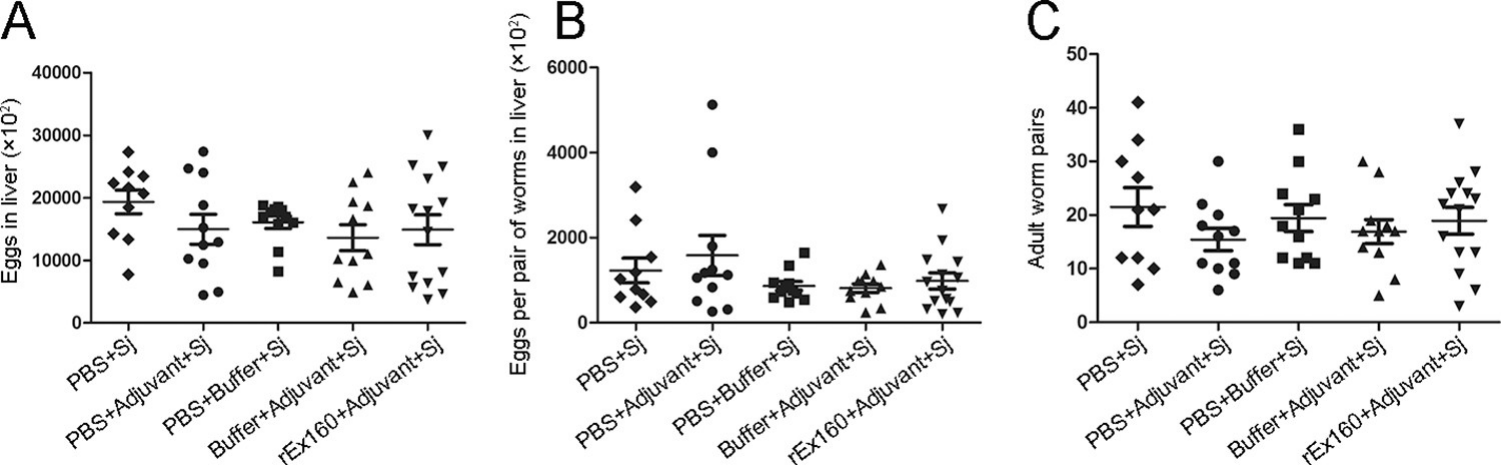
Egg counts is not associated with pathological change in the liver. Rabbits were immunized with PBS, PBS+Adjuvant, PBS+Buffer, Buffer+Adjuvant, or rEx160+Adjuvant and subsequently infected with *S*. *japonicum*. Parts of the rabbit livers were collected, weighed, and digested for egg counting at 8 weeks post-infection. Worm pairs were collected and counted. The total egg number in liver and total egg production number per pair of worms were calculated. (A) The total egg number in liver. (B) The total egg production number per pair of worms. (C) The number of worm pairs in rabbits. Each point represents the number of eggs or worm pairs in a single rabbit. Data were means ± SEM of samples from two independent experiments. (Sj: *S*. *japonicum*).

Some pathological responses in schistosomiasis infection is directly linked to the number of eggs laid by the worms. In order to evaluate the role of egg and worm pair counts in the down-modulation of liver pathology by rEx160 immunization, number of the eggs in the liver and the worm pairs were analyzed. The egg counts and worm pairs were very similar throughout all the groups including the one of the rEx160 immunization (Fig 4). The results indicated that egg load in the liver is not associated with alleviation of the pathological change in the liver by rEx160 immunization.

### rEx160 immunization reduces schistosome egg maturation

**Fig 5:**
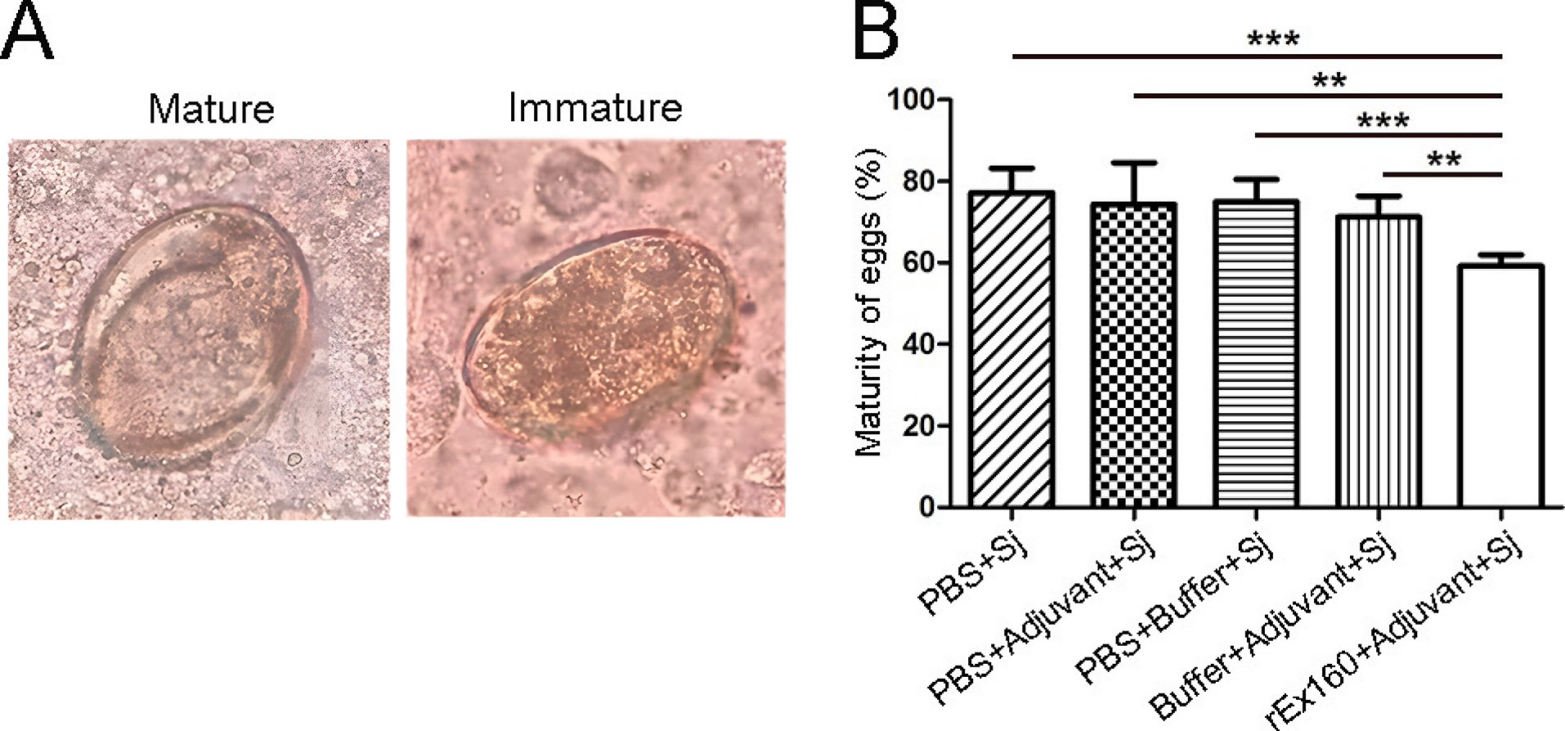
rEx160 immunization reduces schistosome egg maturation. Rabbits were immunized with PBS, PBS+Adjuvant, PBS+Buffer, Buffer+Adjuvant, or rEx160+Adjuvant and subsequently infected with *S*. *japonicum*. Parts of the rabbit livers were collected, weighed, and digested for the evaluation of egg maturation at 8 weeks post-infection. The maturation of eggs was determined under the light microscope and the maturity of eggs was calculated as described in Materials and Methods. (A) Representative photos of mature and immature eggs. (B) Quantification of egg maturation. Data were means ± SEM of samples from two independent experiments. (Sj: *S*. *japonicum*; **P≤0.01, ***P≤0.001).

It was then investigated whether rEx160 immunization reduced schistosome egg maturation. Maturation of the eggs recovered from the liver of rEx160 immunized rabbits were significantly suppressed in comparison to the eggs from the control group rabbits (Fig 5). They are consistent with the previous data that the antibody against rEx160 suppressed the maturation of eggs *in vitro* [12]. As immature eggs are known markedly less antigenic than matured ones [2,5,16], these results provided *in vivo* evidence for the preventive effect of Ex160 specific antibody on egg maturation. Despite the lack of significant similarity between the mRNA of *S*. *japonicum* CD36RP and the mRNA of the primary HDL receptor, scavenger receptor class B member 1 (SR-B1), in rabbits, as well as the absence of significant similarity between the mRNA sequences of *S*. *japonicum* CD36RP and rabbit CD36 (S1 Table), with only 29% similarity between the amino acid sequences of *S*. *japonicum* CD36RP and rabbit CD36 (S1 Fig), we still performed western blotting to investigate whether antibodies induced by rEx160 immunization can bind to rabbit liver proteins, aiming to exclude potential immune cross-reactivity between HDL receptors in rabbit liver and *S*. *japonicum* CD36RP. The results demonstrated that the antibodies induced by rEx160 immunization were unable to bind to proteins in rabbit liver (S2 Fig). Since it remains unclear whether CD36RP in the eggs of the parasite is secreted, potentially inhibiting the effectiveness of rEx160 immunization, we also examined the binding of antibodies induced by rEx160 immunization to proteins in the culture supernatant of *S*. *japonicum* eggs. The results revealed that the antibodies induced by rEx160 immunization were unable to bind to proteins in the culture supernatant of schistosome eggs (S3 Fig). These results suggested that the antibodies generated by rEx160 immunization suppressed egg maturation and alleviated liver pathology in rabbits infected with *S*. *japonicum* by binding to HDL receptor on schistosome eggs.

### Pathology and egg maturation are also reduced after rEx160 immunization in the intestine of the *S*. *japonicum* infected rabbits

**Fig 6:**
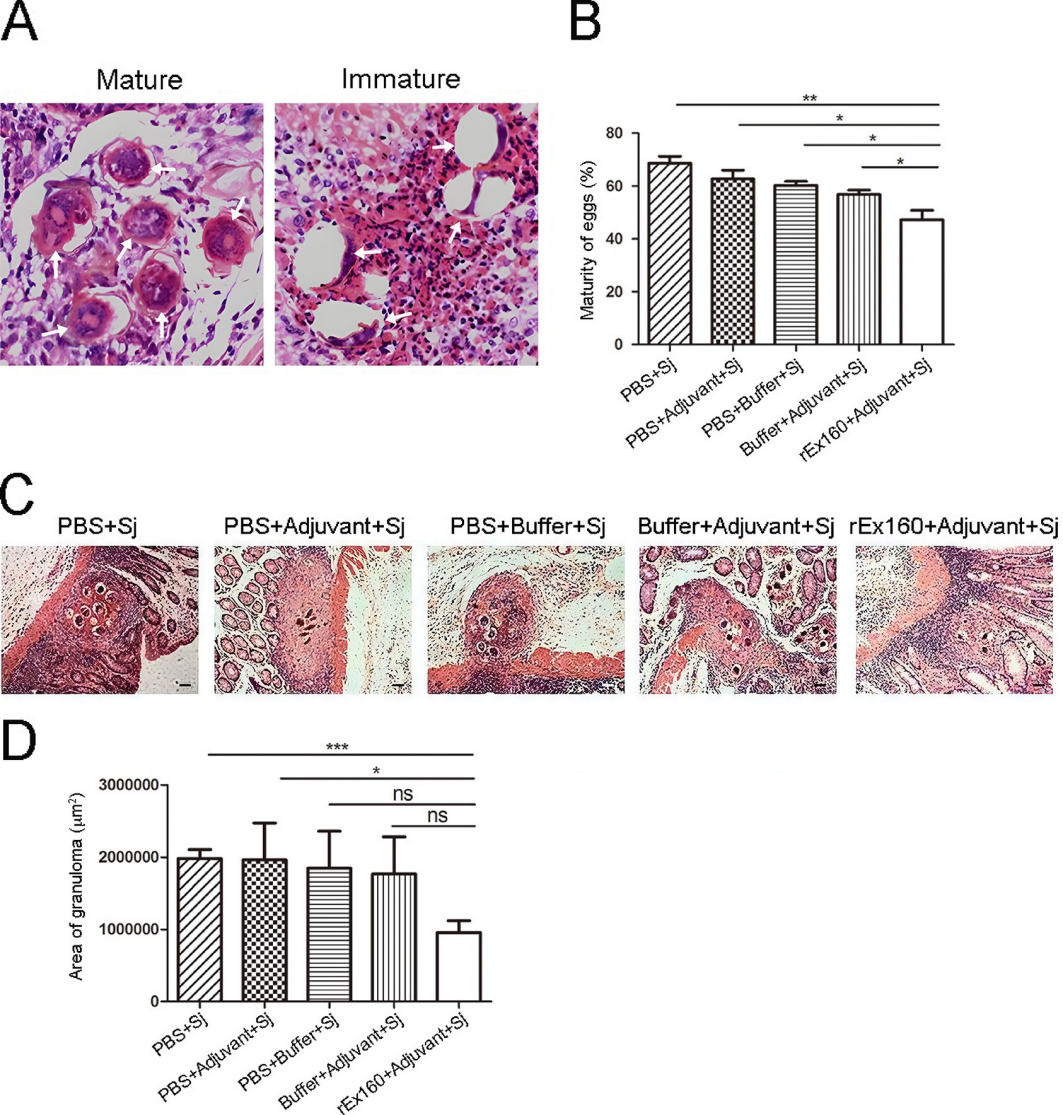
Pathology and egg maturation are also reduced after rEx160 immunization in the intestine of the *S*. *japonicum* infected rabbits. Rabbits were immunized with PBS, PBS+Adjuvant, PBS+Buffer, Buffer+Adjuvant, or rEx160+Adjuvant and subsequently infected with *S*. *japonicum*. The colons were harvested at 8 weeks post-infection and H&E staining was performed in colon sections. The maturation of eggs was determined under the light microscope and the maturity of eggs was calculated as described in Materials and Methods. (A) Representative photos of mature and immature eggs. White arrows indicate mature or immature eggs. (B) Quantification of egg maturation. Measurement of granuloma area was carried out. (C) Representative photos of granulomas with the original magnification of 100× (scale bar = 50 μm). (D) Quantification of granuloma area. Data were means ± SEM of samples from two independent experiments. (Sj: *S*. *japonicum*; *P≤0.05, **P≤0.01, ***P≤0.001).

Maturation of the eggs was also examined in the intestines. As shown in Fig 6A and 6B, maturity of the eggs deposited in the intestine of rEx160 immunized rabbits were significantly lower than the eggs from the control group rabbits. In accordance with the pathological findings in the liver, the level of granulomatous response in the intestine was also reduced after rEx160 immunization, although some of the changes were not statistically significant (Fig 6C and 6D). These results confirmed that rEx160 immunization reduced the egg maturation and subsequently the pathology of tissues.

### Change in serum HDL levels in *S*. *japonicum* infection and the effect of rEx160 immunization in the rabbit model

**Fig 7:**
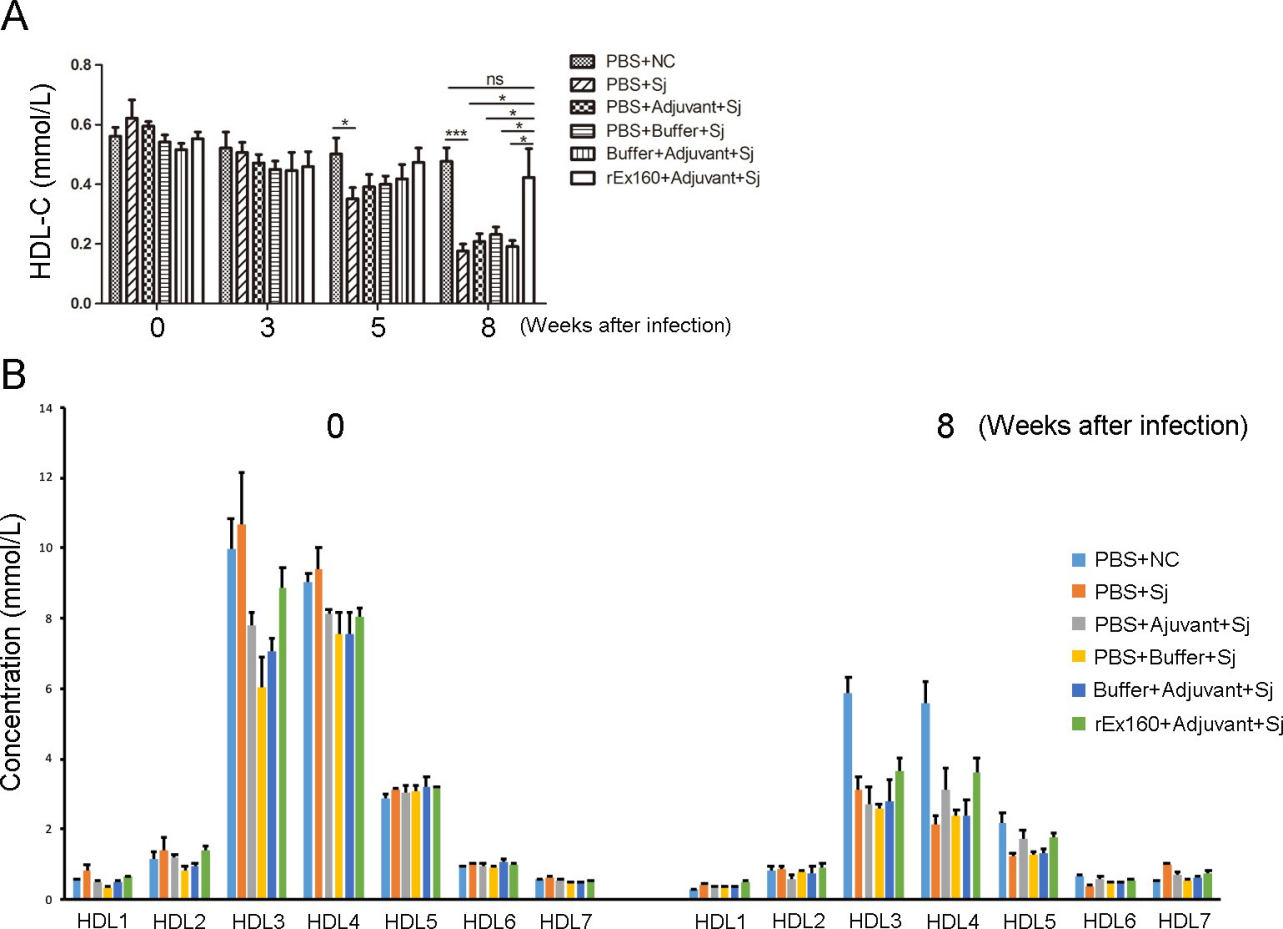
Change in serum HDL levels in *S*. *japonicum* infection and the effect of rEx160 immunization in the rabbit model. Rabbits were immunized with PBS, PBS+Adjuvant, PBS+Buffer, Buffer+Adjuvant or rEx160+Adjuvant and subsequently infected with *S*. *japonicum*. Rabbits injected with only PBS but without infection served as normal control (NC). (A) Serum samples were collected at indicated time points and HDL-C levels were measured. (B) HDL subfraction profile by HPLC analysis of the samples at 0 week and 8 week after infection. Data were means ± SEM of samples from two independent experiments. (NC: normal control; Sj: *S*. *japonicum*; *P≤0.05, ***P≤0.001).

Serum HDL-C levels were determined in the rabbits during the course of *S*. *japonicum* infection experiments. Results showed that significant decrease of serum HDL-C became apparent at 5 weeks of the infection (on week 11), and it further declined after 8-week infection (on week 14) (Fig 7A). Decrease in HDL-C was significantly alleviated by rEx160 immunization in the schistosome infected rabbits (Fig 7A). HDL subfractions were analyzed by HPLC to examine any specific part of HDL are affected in these processes. No specific fraction seems influenced either in the reduction of HDL and or its rescue by rEx160 immunization (Fig 7B). The data are consistent with previous reports that HDL-C significantly decreased at the infection period of egg deposition in *S*. *japonicum*-infected wild-type and CETP-transgenic mice [9]. Since the antibody against rEx160 suppressed the CE uptake by schistosomes *in vitro* [12], these results can be interpreted that HDL-CE uptake by the parasite eggs was blocked by the rEX160 immunization, which leads to suppression of the egg maturation and retards development of the liver complication in *S*. *japonicum*-infected rabbits.

## Discussion

Human schistosomiasis remains as a substantial life-threatening public health problem in many parts of the world. The most serious, intractable and sometimes fatal complication is granulomatosis and fibrosis of the liver caused by the embolized eggs as they are ectopically matured to embryonation [2–4]. Although the current control measures based on mass drug (praziquantel) administration are effective in killing adult schistosome worms [17,18], the sustained pathology due to mature eggs that have already been laid cannot be overlooked [19,20]. Hence, there is an urgent need to develop any egg-targeting strategies against pathology induced by schistosome infection. The data presented here showed that rEx160 immunization offered significant efficacy against egg maturation and led to suppression of hepatic immunoinflammatory process in the *S*. *japonicum*-infected rabbits.

In schistosomiasis, the major vaccine strategy so far has been focused on anti-infection, which is against invading larval stages to prevent or reduce infection [21,22]. Thus, very limited protection in the liver pathology has been achieved because pathology of the disease is largely caused by the host response to the eggs rather than the infecting worms themselves [2,6]. Pathology associated with *S*. *japonicum* infection is a result of granuloma formation surrounding the parasite eggs trapped in the sinusoids of the liver, a process that leads to increased collagen synthesis and severe hepatic fibrosis [2,4,6]. Thus, different strategies, e.g. targeting the eggs, should be considered in anti-pathology vaccination design.

Granuloma formation around schistosome eggs is elicited by soluble egg antigens (SEA) [23,24], and mature eggs are much more antigenic than immature ones [5,16,25]. The granulomatous reaction in *S*. *japonicum*-infected mice liver is very little until the egg displays maturation to miracidium [25]. Studies showed that immature eggs isolated from worms maintained in culture resulted in virtually no granuloma formation when injected intravenously into the pulmonary microvasculature of mice [16]. In addition, intraperitoneal injection of SEA extracted from immature eggs failed to sensitize mice to granuloma formation around the eggs subsequently injected into the pulmonary veins [16]. Thus, the egg maturation is one of key targets to prevent fatal liver complication development in the schistosomiasis.

We discovered that host plasma HDL is a key nutrient source for maturation of the *S*. *japonicum* eggs to miracidia [9] and identified the HDL receptor protein CD36RP to mediate CE uptake from HDL for the egg maturation [12]. Our data further suggested that antibody against the extracellular loop peptide of CD36RP, Ex160, significantly prevented HDL-CE uptake by the *S*. *japonicum* eggs and their the embryonation in *in vitro* culture [12]. Therefore, an initial objective of our study was to determine whether immunization of animals with the peptide Ex160 alleviates liver complication by halting egg maturation. We therefore developed recombinant Ex160 for the purpose of immunization of the animals to be infected with *S japonicum*. We observed some variations in the levels of anti-Ex160 IgG antibodies in rabbit serum at different time points following immunization with rEx160. IgG antibodies typically start to appear 2–3 weeks after primary antigen immunization [26–28]. If there is prior immunity against the antigen, IgGs may appear within several days after secondary antigenic stimulation [26–28]. Therefore, we detected the highest levels of anti-Ex160 IgG antibodies at week 6, two weeks after the third immunization. The half-life of IgG antibodies is approximately 3 weeks [29,30], it probably resulted in a decline in anti-Ex160 IgG antibody levels at week 9. Three weeks after schistosome infection (week 9), adult worms begin to lay a large number of eggs, and Ex160 is expressed in the schistosome eggs. Therefore, we speculate that the re-elevation of anti-Ex160 IgG antibodies occurred two weeks after the start of egg production by the schistosomes (week 11) due to exposure of Ex160 antigen resulting from minimal leakage of Ex160 or a small amount of ruptured eggshells.

In the animal experiments using a rabbit model, examination by H & E and Sirius Red staining of the liver sections revealed that development of granuloma or fibrosis was significantly reduced by rEx160 immunization. The percentage of mature eggs in the liver of the infected rabbit was significantly reduced by the rEx160 immunization clearly indicating that impairing the maturation of schistosome eggs helps reduction of the liver complication. In accordance with the results in liver, the percentage of mature eggs as well as the pathology in the intestine were also reduced by the rEx160 immunization. Our results support the idea that immunization with rEx160 significantly alleviated liver pathology of *S*. *japonicum*-infected rabbits by reducing the maturation of schistosome eggs.

CD36RP is a transmembrane protein, so its localization on the parasite eggs is theoretically on the Von Lichtenberg’s envelope or miracidium, which contains cells [31]. Therefore, we speculate that the anti-Ex160 IgG antibodies induced by rEx160 immunization bind to the cells within the Von Lichtenberg’s envelope or miracidium of the parasite eggs, inhibiting the uptake of host HDL-derived CE by the eggs. To exclude the possibility of the inhibitory effect of rEx160 immunization on the maturation of schistosome eggs being influenced by immune cross-reactivity between CD36RP and the HDL receptors in rabbit liver, we tested the binding between rEx160-immunized rabbit serum and liver proteins from normal rabbits. However, no binding was observed. Furthermore, to rule out the presence of CD36RP in the peripheral blood of rabbits infected with *S*. *japonicum* in the form of secreted proteins, which could potentially interfere with the binding of anti-Ex160 IgG induced by rEx160 immunization to CD36RP on adult worms or eggs, thereby affecting the inhibitory effect of rEx160 immunization on the maturation of schistosome eggs, we tested the binding between rEx160-immunized rabbit serum and proteins in the culture supernatant of *S*. *japonicum* eggs. Similarly, no binding was detected.

The uptake of CE from HDL was demonstrated as essential requirement for maturation of schistosome eggs [9] and the HDL receptor of the parasite CD36RP was shown to mediate this reaction [12,13]. In *in vitro* culture system, the antibody against Ex160 successfully blocked CE uptake from HDL by *S*. *japonicum* and suppressed the maturation of the eggs [12]. It was therefore intended to investigate whether Ex160 immunization could efficiently raise enough antibodies to suppress the uptake of lipids from HDL by *S*. *japonicum* and reduce the maturity of eggs *in vivo* by using rEx160. Our results here clearly demonstrated that rEx160 immunization induced high titers of Ex160-specific antibody and led to suppression of schistosome egg maturation and decrease in development of the liver lesions. A significant prevention of HDL-C reduction was also observed by the immunization with rEx160. No specific HDL subfraction was affected in such alternation of HDL. Small particle subfraction is more susceptible in reduction of HDL in chronic liver diseases [32], so that it is rational to assume that decrease in HDL-C and its rescue by rEx160 immunization is due to consumption of HDL-CE in host blood plasma by the *S*. *japonicum* eggs and its blockade by the treatment.

Our study also showed that the maturation of eggs was not completely suppressed by rEx160 immunization. One possibility for this incomplete effect is shortage of titer of the antibodies. In this study, each rabbit was infected with 100 *S*. *japonicum* cercariae and each *S*. *japonicum* worm pair lays at least 1,000 eggs per day [33]. Raising of the antibodies by the immunization procedure employed in this study may not have been enough to provide adequate protection against the egg-induced liver impairment. It is to be noted that reduced immunogenicity of vaccine may be associated with reduced protection, so that new vaccination strategies may be required to improve the efficacy, including higher vaccine dose, more powerful adjuvants or an alternative delivery route such as microneedle- or nanoparticle-based antigen delivery systems [34–36]. Other possibility is other pathways to detour the HDL receptor for nutrient supply to the eggs. The receptors have been identified for other lipoprotein such as low-density lipoproteins (LDLs) and very low-density lipoproteins (VLDLs) in schistosomes [37–39]. Furthermore, other multiple complex mechanisms are involved in egg maturation such as amino acid and vitamin consumption for embryonation of the eggs in addition to lipid uptake as an important nutritional resource [8].

In summary, our study revealed that immunization with rEx160 protein of *S*. *japonicum* significantly induced anti-Ex160 IgG antibodies in rabbits. Furthermore, immunization with rEx160 protein significantly inhibited the maturation of schistosome eggs in infected rabbits, thereby alleviating hepatic pathology. However, a limitation of this study is that we have not elucidated the exact localization of CD36RP on the eggs, which hinders a deeper exploration of the underlying mechanisms through which rEx160 immunization suppresses egg maturation. Nevertheless, our rEx160 immunization-based pilot study successfully suggested a new egg-targeting strategy intending inhibition of egg maturation by blocking a key nutrient supply is workable for limiting development of the liver pathogenesis after *S*. *japonicum* infection. It provides a starting point for future works to improve the efficacy of vaccination.

## Supporting information

S1 Data Materials and methods relevant to the supplementary table and figures, as well as the optimized nucleotide sequence for Ex160. (DOC)

S1 Table The mRNA sequence similarity alignment. (DOCX)

S1 Fig BLAST alignment of the amino acid sequences of *S*. *japonicum* CD36RP and rabbit CD36. The amino acid sequence similarity alignment between *S*. *japonicum* CD36RP and rabbit CD36 was conducted using NCBI BLAST as described in Materials and Methods (S1 Data). A screenshot of the sequence alignment results is provided.
(TIF)

S2 Fig The binding affinity between liver proteins from normal rabbits and rabbit serum immunized with rEx160. Rabbits liver proteins were isolated as described in Materials and Methods (S1 Data). Western blotting was performed to evaluate the binding affinity between liver proteins from normal rabbits and rabbit serum immunized with rEx160. Results are representative of two experiments with similar results.
(TIF)

S3 Fig The binding affinity between proteins in the culture supernatant of schistosome eggs and rabbit serum immunized with rEx160. Schistosome eggs were cultured *in vitro* and the culture supernatant proteins were isolated as described in Materials and Methods (S1 Data). Western blotting were performed to evaluate the binding affinity between proteins in the culture supernatant and rabbit serum immunized with rEx160 (left). Ponceau S staining was employed to visualize all proteins (right). Results are representative of two experiments with similar results.
(TIF)
