# Supplementary material for: Vaccination against the HDL receptor of S. japonicum inhibits egg embryonation and prevents fatal hepatic complication in rabbit model
Source: PLoS Negl Trop Dis. 2023 Nov 29;17(11):e0011749. doi: 10.1371/journal.pntd.0011749 (PMC10686426; doi:10.1371/journal.pntd.0011749)
Supplement: S1 Data — (DOC) [file pntd.0011749.s001.doc]

**Supporting Information**

**Vaccination against the HDL Receptor of *S. japonicum* Inhibits Egg Embryonation and Prevents Fatal Hepatic Complication in Rabbit Model**

Jifeng Zhu^1†^, Lina Zhang^2†^, Zechao Xue^1^, Zilüe Li^1^, Chun Wang^1^, Fanyan Chen^1^, Yalin Li^1^, Yang Dai^3^, Yonghua Zhou^3^, Sha Zhou^1^, Xiaojun Chen^1^, Kuniko Okumura-Noji^4^, Rui Lu^4^, Shinji Yokoyama^4^*, and Chuan Su^1^*

**Materials and Methods**

***In vitro* cultivation of schistosome eggs.** The pairs of schistosome adult worms were recovered from the portal vein of the *S. japonicum* infected rabbits and cultured in 6-well culture plates in RPMI 1640 medium supplemented with 10% FBS and 1% penicillin/streptomycin in 5% CO2 atmosphere. After 3 days, the worms were removed, and the eggs were cultured in the same plate until they reached maturity. The culture supernatant was collected for the extraction of supernatant proteins.

**Extraction of liver proteins and culture supernatant proteins.** Partial liver tissues were collected from normal rabbits and sera were collected from rabbits infected with *S. japonicum* for 8 weeks. The liver tissues were homogenized in RIPA lysis buffer containing protease inhibitors. The homogenate was then subjected to centrifugation at 13,800 × g for 10 minutes, and the resulting supernatant was collected as the liver protein samples. Extraction of culture supernatant proteins was performed using a commercial culture supernatant protein extraction kit (Solarbio, Beijing, China) according manufacturer's instructions.

**Western blotting analysis.** Proteins isolated from liver tissues or culture supernatants separated by SDS-PAGE were transferred to a polyvinylidene difluoride membrane (Whatman Inc., Florham Park, NJ, USA). The membrane was blocked with 5% skimmed milk in PBS+0.1% Tween 20 and probed with rabbit serum immunized with rEx160. The blots were visualized using the Pierce ECL Plus Western Blotting Substrate (Thermo Fisher Scientific), detected by the ChemiDoc Touch Imaging System (Bio-Rad Laboratories, CA, USA). For the detection of culture supernatant protein samples, prior to blocking with 5% skimmed milk, the PVDF membrane was first stained with Ponceau S (Beyotime, Shanghai, China).

**Alignment analysis with NCBI BLAST.** The alignment of mRNA sequences and protein amino acid sequences was performed using NCBI BLAST (https://blast.ncbi.nlm.nih.gov/Blast.cgi).For the alignment of mRNA sequences, “Align two or more sequences” function of NCBI Nucleotide BLAST was used to perform the alignment of mRNA sequences for similarity comparison. The program was optimized for somewhat similar sequences (blastn). For the alignment of protein amino acid sequences, “Align two or more sequences” function of NCBI Protein BLAST was used to perform the alignment of amino acid sequences for similarity comparison.

**Full-length optimized nucleotide sequence of Ex160**

GGTAGCGACGGCACCTTCTTTCACAGCTTCCTGACCAAGTACGACAAACCGTATGTGTTTGCGAGCGATATTTGCCGTAGCCTGCAGTTCTACACCGAGAGCATCGACAAGCTGCACAACCTGCCGGTTCTGAAACTGACCCCGATGCTGGATACCTTTAAGAGCCCGAAATACTATGAGAAGAACCGTGGTTTCTGCCTGAACTGGCCGAACTGCTATGAAGACGGTGTGCTGGATATGAGCAGCTGCCAGCCGGGTGCGCCGATTGTGGTTAGCCAACCGCACTTTCTGAACGCGAACAAGACCTACCAGGACGCGGTTGATGGCATGTATCCGACCAACGAGATGAACACCGTGATCTACGTTGAACCGAACACCGGCAGCATCATTAAAGCGCAGAAGAAAATCCAAATTAACATCCTGGTGAAGAACGATACCACCTTCAAACAACTGGCGAACATTAGCACCACCCTGCTGCCGTAA

**Figure legends**

**S1 FIG. BLAST alignment of the amino acid sequences of *S. japonicum* CD36RP and rabbit CD36.**

The amino acid sequence similarity alignment between *S. japonicum* CD36RP and rabbit CD36 was conducted using NCBI BLAST as described in Materials and Methods. A screenshot of the sequence alignment results is provided.

**S2 FIG. The binding affinity between liver proteins from normal rabbits and rabbit serum immunized with rEx160.**

Rabbits liver proteins were isolated as described in Materials and Methods. Western blotting was performed to evaluate the binding affinity between liver proteins from normal rabbits and rabbit serum immunized with rEx160. Results are representative of two experiments with similar results.

**S3 FIG. The binding affinity between proteins in the culture supernatant of schistosome eggs and rabbit serum immunized with rEx160.**

﻿Schistosome eggs were cultured *in vitro* and the culture supernatant proteins were isolated as described in Materials and Methods. Western blotting were performed to evaluate the binding affinity between proteins in the culture supernatant and rabbit serum immunized with rEx160 (left).  Ponceau S staining was employed to visualize all proteins (right). Results are representative of two experiments with similar results.
