# Supplementary material for: Vaccination against the HDL receptor of S. japonicum inhibits egg embryonation and prevents fatal hepatic complication in rabbit model
Source: PLoS Negl Trop Dis. 2023 Nov 29;17(11):e0011749. doi: 10.1371/journal.pntd.0011749 (PMC10686426; doi:10.1371/journal.pntd.0011749)
Supplement: S1 Table — (DOCX) [file pntd.0011749.s002.docx]

S1 Table The mRNA sequence similarity alignment

| Names of the aligned sequences | Results of BLAST analysis |
| --- | --- |
| Rabbit SR-B1 & *S. japonicum* CD36RP | No significant similarity found |
| Rabbit CD36 transcript variant X1 & *S. japonicum* CD36RP | No significant similarity found |
| Rabbit CD36 transcript variant X2 & *S. japonicum* CD36RP | No significant similarity found |
| Rabbit CD36 transcript variant X3 & *S. japonicum* CD36RP | No significant similarity found |
| Rabbit CD36 transcript variant X4 & *S. japonicum* CD36RP | No significant similarity found |
| Rabbit CD36 transcript variant X5 & *S. japonicum* CD36RP | No significant similarity found |
| Rabbit CD36 transcript variant X6 & *S. japonicum* CD36RP | No significant similarity found |
| Rabbit CD36 transcript variant X7 & *S. japonicum* CD36RP | No significant similarity found |

NCBI reference sequence Nos.: Rabbit SR-B1, NM_001082788.1; Rabbit CD36 transcript variant X1, XM_002712016.4; Rabbit CD36 transcript variant X2, XM_008258296.3; Rabbit CD36 transcript variant X3, XM_008258298.3; Rabbit CD36 transcript variant X4, XM_008258297.3; Rabbit CD36 transcript variant X5, XM_008258299.3. Rabbit CD36 transcript variant X6, XM_008258301.3; Rabbit CD36 transcript variant X7, XM_008258300.3; *S. japonicum* CD36RP, AY496973.1.
