# Supplementary figures and images for: Vaccination against the HDL receptor of S. japonicum inhibits egg embryonation and prevents fatal hepatic complication in rabbit model
Source: PLoS Negl Trop Dis. 2023 Nov 29;17(11):e0011749. doi: 10.1371/journal.pntd.0011749 (PMC10686426; doi:10.1371/journal.pntd.0011749)

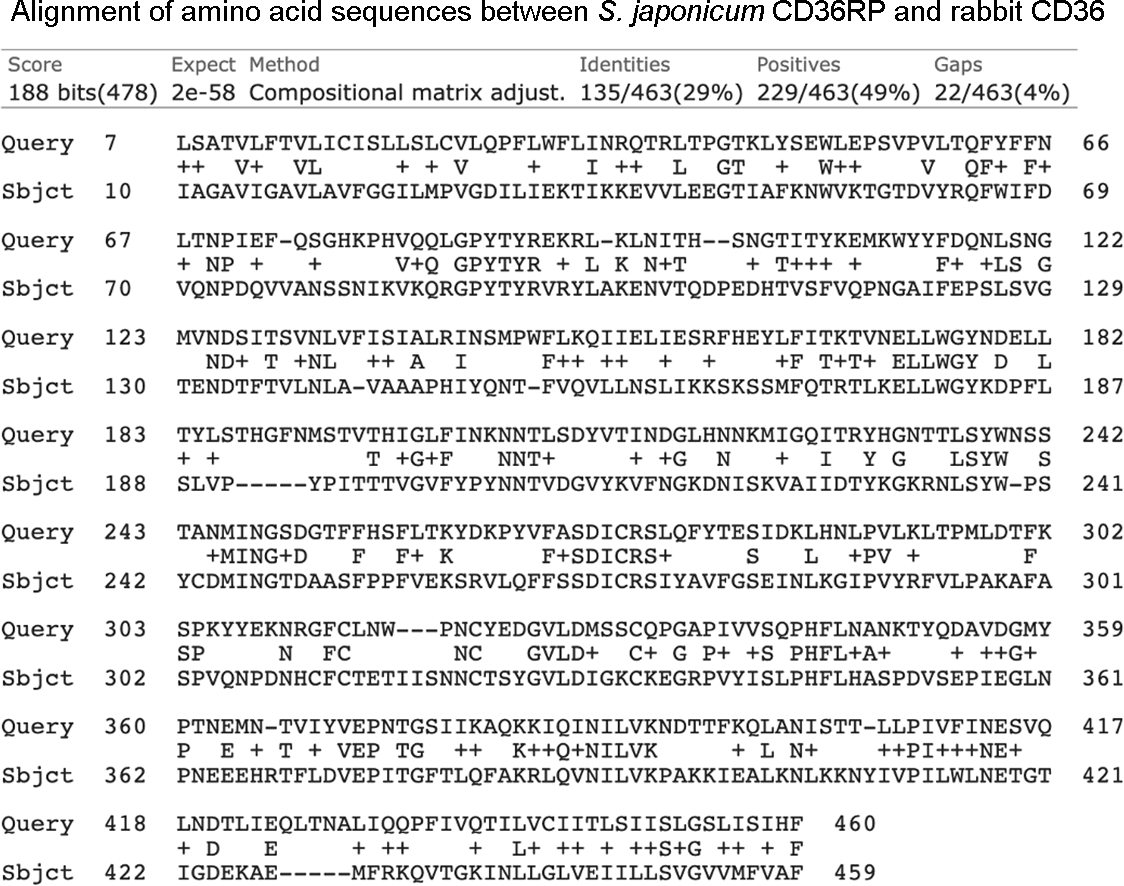

Supplement: S1 Fig — The amino acid sequence similarity alignment between S. japonicum CD36RP and rabbit CD36 was conducted using NCBI BLAST as described in Materials and Methods (S1 Data). A screenshot of the sequence alignment results is provided. (TIF) [file pntd.0011749.s003.tif]

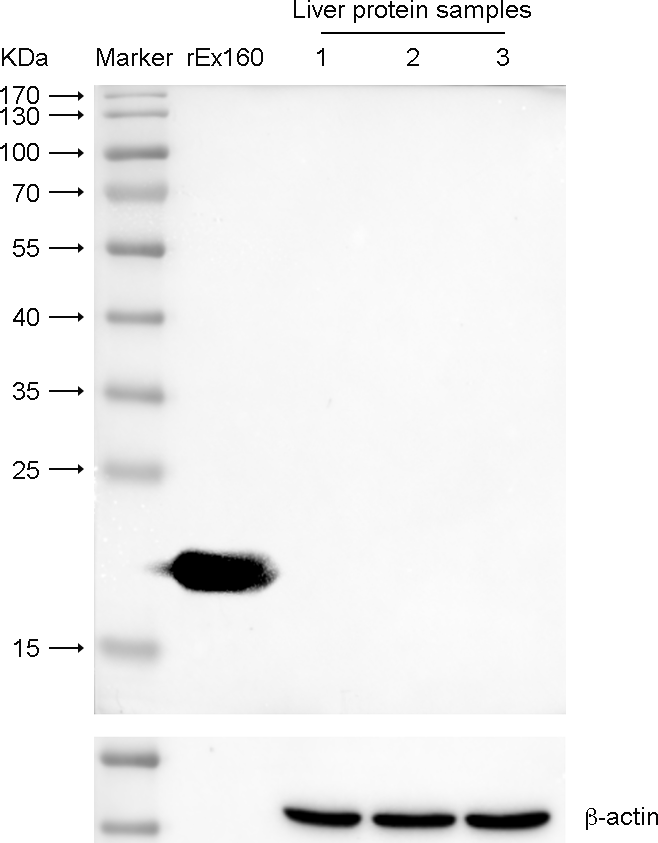

Supplement: S2 Fig — Rabbits liver proteins were isolated as described in Materials and Methods (S1 Data). Western blotting was performed to evaluate the binding affinity between liver proteins from normal rabbits and rabbit serum immunized with rEx160. Results are representative of two experiments with similar results. (TIF) [file pntd.0011749.s004.tif]

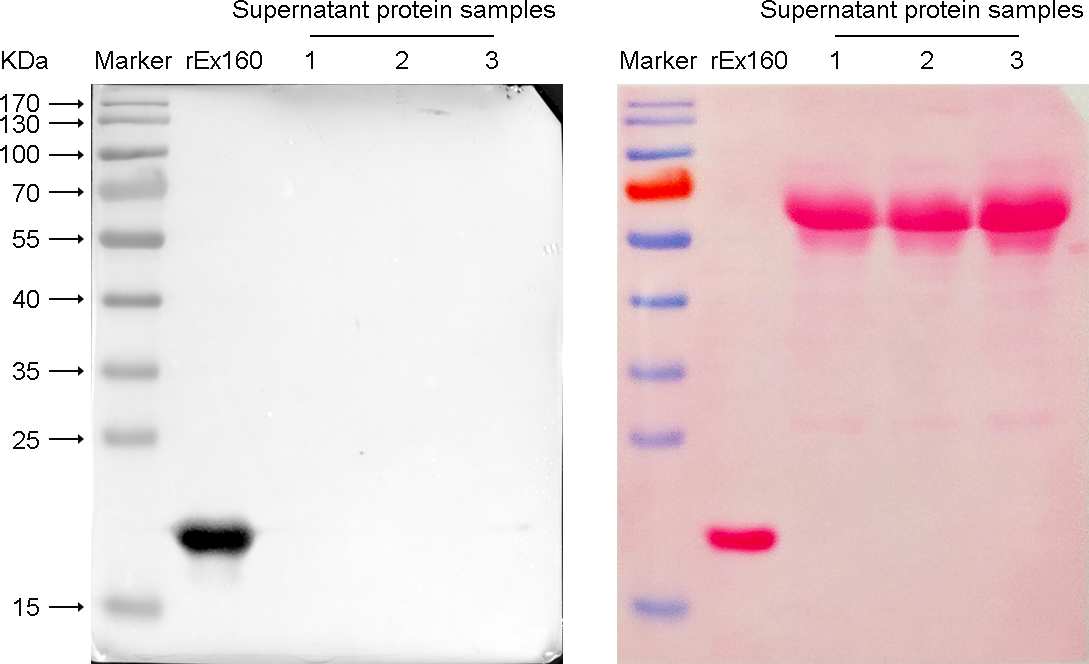

Supplement: S3 Fig — Schistosome eggs were cultured in vitro and the culture supernatant proteins were isolated as described in Materials and Methods (S1 Data). Western blotting were performed to evaluate the binding affinity between proteins in the culture supernatant and rabbit serum immunized with rEx160 (left). Ponceau S staining was employed to visualize all proteins (right). Results are representative of two experiments with similar results. (TIF) [file pntd.0011749.s005.tif]
